# The Musical Self-Concept of Chinese Music Students

**DOI:** 10.3389/fpsyg.2016.00776

**Published:** 2016-05-27

**Authors:** Suse Petersen, Marc-Antoine Camp

**Affiliations:** School of Music, Lucerne University of Applied Sciences and ArtsLucerne, Switzerland

**Keywords:** musical self-concept, self-concept types, Chinese music students, higher music education, musical ability

## Abstract

The relationship between self-concept and societal settings has been widely investigated in several Western and Asian countries, with respect to the academic self-concept in an educational environment. Although the musical self-concept is highly relevant to musical development and performance, there is a lack of research exploring how the musical self-concept evolves in different cultural settings and societies. In particular, there have been no enquiries yet in the Chinese music education environment. This study’s goal was the characterization of musical self-concept types among music students at a University in Beijing, China. The Musical Self-Concept Inquiry—including ability, emotional, physical, cognitive, and social facets—was used to assess the students’ musical self-concepts (*N* = 97). The data analysis led to three significantly distinct clusters and corresponding musical self-concept types. The types were especially distinct, in the students’ perception of their musical ambitions and abilities; their movement, rhythm and dancing affinity; and the spiritual and social aspects of music. The professional aims and perspectives, and the aspects of the students’ sociodemographic background also differed between the clusters. This study is one of the first research endeavors addressing musical self-concepts in China. The empirical identification of the self-concept types offers a basis for future research on the connections between education, the development of musical achievement, and the musical self-concept in societal settings with differing understandings of the self.

## Introduction

Musical self-concept, which plays an important role in a person’s musical development and achievement, has received increasing academic interest in the recent past (e.g., Sanders and Browne, 1998; Ruismäki and Tereska, 2006; Buchborn and Painsi, 2010; Spychiger, 2012). However, almost no research has been conducted on the emergence of the musical self-concept in different cultural settings. There is similarly a lack of research regarding different dimensions of the musical self-concept: scholars have explored the general self-concept (e.g., Shavelson et al., 1976; Marsh and Shavelson, 1985; Marsh and Martin, 2011), or have chosen as center stage of their research non-musical facets such as the academic self-concept (with sub-dimensions like the verbal or math self-concept) and its relation to achievement and motivation (e.g., Marsh, 1993; Marsh and Hau, 2004; Marsh and Martin, 2011; McInerney et al., 2012). One attempt to investigate different dimensions of the musical self-concept was undertaken by Vispoel (1996), who developed the *Arts Self-Perception Inventory* (ASPI), with subscales in four artistic domains. One of these, music, was measured with the *Music Self-Perception Inventory* (MUSPI; a short version, the MUSPI-S, was published recently in Morin et al., 2015).

While the MUSPI concentrates on a person’s musical skills, the *Musical Self-Concept Inquiry* (MUSCI), developed by Spychiger et al. (2009), was not only designed to measure perceived skills and abilities, but additional dimensions. The inquiry assesses emotional, communicative, technical, physical, and spiritual aspects in relation to music, as well as aspects of an ideal and changing musical self (Spychiger, 2012). By this conceptual extension, it is assumed that an individual’s relation to music encompasses much more than just potentially existing skills. It consists of one’s varying roles as performer, teacher, learner, facilitator, or recipient of music, and touches many facets of life. In this way, the MUSCI takes into account the fact that context as well as cultural values are (re-)produced in music education and musical activities, which in turn influence musical skills^1^ (Hallam, 2011) and musical self-perceptions^2^ (Hargreaves et al., 2002, 2003). Students’ families and teachers influence their musical self-concepts through their own relationships to music, their own musical self-concepts and, particularly in the case of family, as carriers of cultural values (Wang, 2006). Moreover, as music students will usually teach others at some point in their life, they will influence their pupils’ self-concepts. Hence, the investigation of students’ musical self-concept in its multi-dimensionality relates to environmental settings and bears a future-directed impact.^3^ Furthermore, as the self-concept moderates students’ activities, thinking, and behavior (Oyserman et al., 2011), it influences a person’s relationship to music and consequently his or her musical learning at university. Due to the increasing number of music students studying abroad, such an assessment of the musical self-concept becomes an important task (Garbati and Rothschild, 2016), in particular because it supports reciprocal understanding between young Chinese musicians studying in another country and their teachers.

The MUSCI (Spychiger et al., 2010) was developed in a German and English version and used in German speaking countries. This study is the first application of the MUSCI in an Asian context and aims to:

(1) Examine the musical self-concept in a Chinese setting
(2) Characterize musical self-concept types among Chinese university music students.

Our undertaking involved two methodological challenges:

– Appropriateness of measuring tool: Yeung (2005) has pointed to possible difficulties arising from the use of self-concept scales in a Chinese setting due to the Western context in which the scales were originally developed and tested. Certain expressions, formulations, or syntaxes could lead to misconceptions by the study participants. Items might have to be excluded if they cannot be interpreted meaningfully in an Asian or Chinese context.
– Interpretation of results: To understand differences between results obtained in Western and Chinese settings, references to cultural values in each environment are necessary. In the case of music education in China, Chinese government and its ideologies have been a determining force in recent decades, however, values from outside of China have also been incorporated, affecting, for example, the songs and musical styles used in music lessons (e.g., the predominance of European art music traditions in music conservatories; Ho and Law, 2004). Regarding general environmental settings, Asian cultures, including the Chinese, have often been said to be traditionally “collectivistic” societies (e.g., Chen, 2000) in contrast to the “individualistic” focus of Western cultures. Accordingly, a person’s self-concept shows “collectivist” or “individualistic” orientations (Heine, 2001). Chinese learners have been perceived as being influenced by Confucian ideals about life in general—for example, embracing the value of social harmony and displaying the tendency toward emotional regulation (Bond, 1993), and learning in particular—with importance placed on behaviors such as obedience toward authority figures or self-cultivation and self-enhancement (Tweed and Lehman, 2002; Li, 2005; Huang, 2012; Phillipson, 2013; Wang, 2013). This was seen both as a factor in the successes of Chinese students, and as a reason for the criticized—but often also falsely attributed—tendency toward rote learning and for lack of creativity (Brand, 2001). Cultural aspects, or the differences between Asian and Western cultures, also served as an explanation for results showing that Chinese music education students’ self-esteem is significantly lower than that of Western students—without concluding that the framework of self-esteem is necessarily the same (Brand, 2004).

The discussion section points to cultural values in language and interpretation, considering, however, that the Chinese setting today is simultaneously different from Western settings and similar to it due to processes of internationalization.

## Materials and Methods

This study represents the first use of the MUSCI in China. It therefore included a principal component analysis in order to re-test the items’ factor loadings. Based on the assumption that the measurements of factor affiliations would differ slightly from the MUSCI, the intention was to adapt the scales or factors according to the possible distinctions between the European data [collected during the development of the MUSCI by Spychiger et al. (2010)] and Chinese data. Indeed, Spychiger et al. (2010, p. 26) suggested that the constructs of the musical self-concept might appear differently depending on the context. The clusters measured in our study have been compared to the results and types identified by Spychiger et al. (2010). As that study served to develop the MUSCI scales, and our study only included music students in Beijing, validation studies with larger samples—of musicians and non-musicians in different cultures and from a cross-cultural perspective—are still pending.

### Participants

Participants in the questionnaire survey were undergraduate and graduate music education students (*N* = 97) at a university in Beijing, PR China. The questionnaires were distributed in paper-and-pencil form at the beginning of lectures, and the students were given as much time as necessary for filling out the questionnaire. Participation was voluntary and consent was sought in advance. The students were informed verbally and in writing that their data would be kept confidential and stored anonymously. The study was approved by the research committee of the School of Music of Lucerne University of Applied Sciences and Arts.

### Materials

The first part of the survey instrument included the MUSCI developed by Spychiger et al. (2010, list of items in supplementary material). Participants indicated their level of agreement with the items on a 4-point Likert-scale. Subscales of the MUSCI are the following:

(1) Mood Management (six items)
(2) Community (four items)
(3) Technique and Information (four items)
(4) Musical Ability (ten items)
(5) Movement and Dance (five items)
(6) Spirituality (four items)
(7) Ideal Musical Self (five items)
(8) Adaptive Musical Self (four items)

These dimensions were measured with items answerable by musicians and non-musicians. Additional subscales for musicians only assess the following dimensions:

(9) Musical Communication (six items)
(10) Performance and Ambition (six items)
(11) Emotional Involvement (five items)
(12) Spiritual Experiences (four items)

The MUSCI was originally developed in German and translated into English by Spychiger (in press). We used a slightly adapted version, presenting all items in English and Chinese. The items were discussed thoroughly with native speakers of both languages and constantly compared to the original items in German and English. Given the use of the existing scale in English and the very slight rephrasing of items, no pilot study was undertaken. The English–Chinese version of the MUSCI will be revised according to our results and the adapted scales will be addressed in the discussion section.

The second part of the questionnaire included questions about the students’ musical upbringing (e.g., familial affiliation with musical activity), their educational level and career goals, their studies and daily musical practice, and further sociodemographic details. These questions were presented either as closed-ended questions (e.g., gender) or as semi-closed-ended questions (e.g., instrument played, or career goal).

## Results

### Descriptive Statistics

Ninety-seven questionnaires were returned and missing data was excluded listwise when MUSCI items were not answered. The final sample consisted of 72 records and included 61 female students (85%) and 11 male students (15%). The students were 18–27 years old (*M* = 20.71, Mdn = 20, *SD* = 2.22, missing = 1). 38 students (53%) were 18–20 years old, 24 (33%) were 21–24 years old, and 6 (8%) were older than 24. All participants were of Chinese nationality.

Participants’ main instruments were distributed across piano (*n* = 31), voice (*n* = 20), erhu (*n* = 6), guzheng (*n* = 5), clarinet (*n* = 2), dizi (*n* = 2), pipa (*n* = 2), and—with one person playing each instrument—accordion, drums, “national instrument” (probably guzheng or pipa), and sanxian. A Western instrument (accordion, clarinet, drums, and piano) was therefore played by 49% or 35 students; 24% or 17 students played a Chinese instrument (dizi, erhu, guzheng, pipa, and sanxian); and 28% or 20 students were studying singing. The age at which the students had begun playing their instrument ranged from 3 to 18 years old (*M* = 8.3, *MD* = 7, *SD* = 4.31). 48% of the students were 6 years old or younger, 26% were 7 to 10 years old, and 24% were 11 years or older. At the time of questioning, the students’ practice hours per day differed notably and ranged from 1 to 7 h. Most of the students practiced for 2 h per day (*M*_o_ = 2). After grouping the practice hours, almost half of the students practiced for 2.5 h per day or less (*n* = 34, 47%), 33 students (46%) practiced 3 to 4 h, and only a minority of five students (7%) practiced more than 4 h per day.

Fifty-six of the 72 evaluable questionnaires (78%) were filled out by undergraduates and 16 (22%) by graduate students. Sixteen of the undergraduate students were in the 1st year, 19 in the 2nd year, and 21 in the 3rd year of the bachelor’s program (respectively, 22, 26, and 29% of all students). Fourth year undergraduates could not be included; they were absent at the time of our investigation because their term dates differed from those of the other undergraduates.

A majority of the students grew up in a household where neither of the parents were musically active at a professional or amateur level (*n* = 43, 60%), while 19 students had at least one parent who played an instrument (26%), and in 10 cases (14%) both parents were either professional or amateur musicians.

The career goals and professional aims of the students varied, with 46 individuals aiming at a teaching career (64%), 11 students (15%) planning to work as performers, and 7 students (10%) looking for a combination of performing and composing, conducting, or teaching. Two students (3%) intended to work as composers, and one student (1%) planned to work as translator. Five students (7%) did not report any professional aims.

### Data Analysis

To test if the variables of the MUSCI met normal distribution criteria necessary for further investigation, skewness and kurtosis were examined. All variables were below the measures of 3 for skewness and 8 for kurtosis, and normal distribution could be assumed (Kline, 1998).

As the MUSCI was translated into Chinese and the sample differed fundamentally from former samples, a principal component analysis was repeated. Spychiger et al. (2010) conducted separate analyses for the first (suitable for musicians and non-musicians) and second (suitable only for musicians) parts of the MUSCI. By contrast, the results discussed below are based on an analysis with all items. This decision was made due to the difficulty of interpreting the factors of separate analyses for both parts. As the topics of the scales in both parts are, to a certain degree, similar (e.g., spirituality and spiritual experience; community and musical communication), it was assumed that more meaningful factors would result if all items were included. Content-wise, the factors of our analysis mirror the original scales quite well and are by and large also suitable for non-musicians.

A principal component analysis (varimax rotation with Kaiser normalization) with the original number of 12 factors of the MUSCI was carried out. Compared to the 12 factors of the MUSCI, our data displays a different factor solution with a lower number of factors. Eight factors could be meaningfully identified and related to the original MUSCI dimensions—with similar topics from the two parts of the questionnaire falling together. Factors which could not be interpreted sensibly (for example, due to items with different themes), and items which had loadings <0.4 or on more than one factor were excluded from further analysis.

A second principal component analysis with a fixed number of 8 factors was carried out and confirmed the eight factor solution. The Kaiser–Meyer–Olkin measure of sampling adequacy (*MSA*) resulted in the reasonable measure of KMO = 0.744 and Bartlett’s Test of Sphericity was significant (Sig. = 0.000). The eight factors had eigenvalues >1 which in combination explained 69.827% of the variance. A further measure to determine the suitability of the data for principal component analysis is the *MSA* (Weiber and Mühlhaus, 2010). *MSA* measures were >0.6, except for items from the original MUSCI dealing with the topic of dance and movement (*MSA* of item 13 = 0.377, *MSA* of item 20 = 0.381, and *MSA* of item 33 = 0.460) and the items concerning community and communication aspects (*MSA* of item 36 = 0.408 and *MSA* of item 43 = 0.381). All items except one (item 41) had loadings >0.5 on one of the factors (lower loadings were suppressed). Item 41 was excluded from further analysis. No loadings >0.5 on more than one factor occurred. Although the movement/dance and community/communication items should be excluded due to insufficient *MSA* measures, the authors decided to keep these items as the internal validity of these scales proved to be satisfactory (see **Table 1**) and the items correspond with the original scales of the MUSCI.

**Table 1:** Reliability statitics.

Scale	Cronbach’s alpha	Number of items
I Achievement and Ambition	0.919	11
II Mood Management	0.846	5
III Ability and Expertise	0.715	4
VI Technique and Information	0.731	3
V Dance	0.794	3
VI Rhythm and Movement	0.881	2
VII Spiritual Experiences	0.716	2
VIII Community and Communication	0.701	2

The eight factors resulting from the principal component analysis are not exactly the same as in the MUSCI, with respect to the item assignment, but they are theoretically plausible and content-wise they are similar to the original MUSCI scales. This will be elaborated in the discussion section after the following display of the item assignment:

(1) The first factor was labelled **Achievement and Ambition** and includes 11 items about the striving for high musical skills and knowledge:
   • *2 It appeals to me to make the most of my musical ability.*
   • *9 I would like to have higher musicianship.*
   • *22 I have no musical talent.*
   • *28 I regret that I am not more musically creative.*
   • *32 I feel that I could have become a great musician.*
   • *42 I would like to have more knowledge of the technical features and options in music.*
   • *44 I am capable of achieving the musical goals that I have set.*
   • *46 I am musically ambitious.*
   • *47 I love applause.*
   • *49 I take advantage of any opportunity to advance my musical ability.*
   • *58 I am proud of my musical skills.*

(2) The second factor was labeled **Mood Management** as the five items deal with the effect music had on the mood of the participants:
   • *1 Music can carry me away from everyday life.*
   • *4 I can purposefully influence my mood through music.*
   • *21 With music I can forget my sorrows.*
   • *30 I can relax with music.*
   • *34 Music helps me to cope with stress.*

(3) The third factor was labeled **Ability and Expertise** and includes four items about the participants’ perceived musical skills and competences:
   • *8 My musical ability is above average.*
   • *12 I have the ability to teach other people about music.*
   • *15 I easily hear harmonies and pick out voices.*
   • *35 I am an expert as regards certain musical styles.*

(4) The fourth factor was labeled **Technique and Information** with three items about theoretical and technical music-related knowledge:
   • *11 I am interested in how musical instruments function.*
   • *16 The technical options to edit music are fascinating to me.*
   • *29 I am concerned with the question of how music is produced.*

(5) The fifth factor was labeled **Dance** and includes three items about dancing:
   • *13 I passionately love to dance.*
   • *20 Dancing satisfies my need for physical movement.*
   • *33 I avoid dancing since I don’t dance well.*

(6) The sixth factor was labeled **Rhythm and Movement**, including two items about moving the body to the rhythm of music:
   • *10 When I hear music, I start to move my body to the rhythm.*
   • *39 I easily move to the rhythm of music.*

(7) The seventh factor, with two items, pointed to a contact with the divine and enabling of prayer through music. It was therefore labeled **Spiritual Experiences**:
   • *51 For me, making music is a special kind of prayer.*
   • *57 I make music in order to feel the divine.*

(8) The eighth factor was labeled **Community and Communication**, as the two items deal with the social component of music:
   • *36 I go to musical events in order to meet people.*
   • *43 I play music in order to communicate with other people.*

The adapted version of the MUSCI will henceforth be labeled MUSCI-CN. In Supplementary Table S1 in the supplementary material, the new factor assignment of the items is displayed with a listing of their original factor assignment. Three MUSCI scales and their items could not be confirmed at all, namely the scales Adaptive Musical Self, Emotional Involvement, and Spirituality. The scales Musical Communication and Community “lost” four items and three items, respectively, and were combined into the two-item scale Community and Communication. In the discussion section we come back to this result.

Reliability analyses indicated a satisfactory reliability for all scales (factors), with Cronbach’s Alpha between 0.701 and 0.919 (**Table 1**). The inter-item correlations (IIC) were >0.3 and the corrected item-total correlations (CITC) >0.5 for all items except for two items of the scale *Ability and Expertise*: items 12 and 15, with a slightly lower CITC of 0.478 and 0.492, respectively. However, these items were retained due to their content consistency with the scales and a lower Cronbach’s Alpha of the corresponding scale if excluded.

**FIGURE 1:**
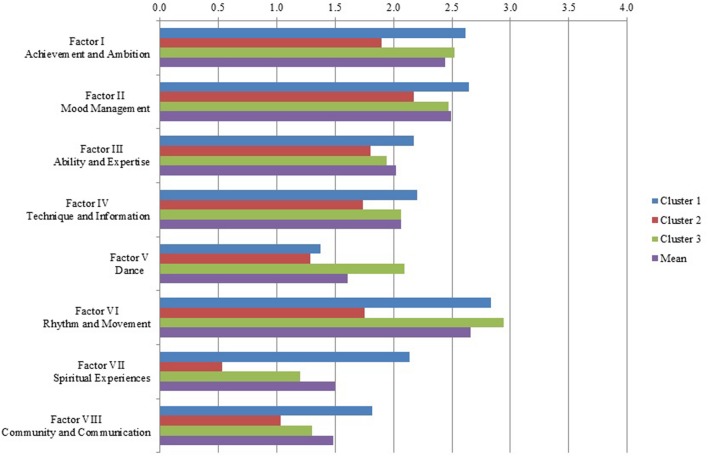
**Manifestation of factor indices**.

New variables with the factor means (factor indices) were computed and a hierarchical cluster analysis (Ward Linkage) was carried out, which is suitable for small sample sizes. The factor indices (as opposed to the factor measures after principal component analysis) were chosen because these measures align with the 4-point Likert-scale and can be better illustrated graphically (**Figure 1**). The interpretation of the dendrogram pointed to a three-cluster-solution. A four-cluster-solution could have also been justified in the dendrogram. However, a relevant increase of the coefficients in the agglomeration schedule table was observed after the 68th fusion stage, also indicating a three-cluster-solution. Another cluster analysis with three clusters was carried out and the cluster membership of the cases was retained as additional variables [cluster 1: *n* = 33 (45.8%); cluster 2: *n* = 14 (19.4%); cluster 3: *n* = 25 (34.7%)].

To test if the clusters differ significantly and, if so, which factors influence the differentiation, a discriminant analysis was executed. The canonical correlation coefficients, showing how well the groups can be separated, were satisfactory (0.866 and 0.734) with eigenvalues of 3.012 and 1.170 for functions 1 and 2, respectively. Wilks’ Lambda was highly significant for the two discriminant functions (*p* < 0.001). The standardized canonical discriminant function coefficients pointed to the highest influence on group membership by factors I, VI, and VII for function 1, and factors I, III, V, VI, and VII for function 2, indicating a high influence through factors I, VI, and VII. The tests of equality of group means (testing if the groups differ significantly regarding the factor variables) were highly significant (*p* < 0.001) for factors I, V, VI, VII, and VIII; very significant for factor II (*p* < 0.01); and significant on a lower significance level (*p* < 0.05) for factor III. Only the test of equality of group means for factor IV was not significant (*p* = 0.066). The classification results showed that 90.3% of the original grouped cases were correctly classified. A cross-validation with the stepwise method led to 88.9% of the cross-validated grouped cases being correctly classified, a result validating the discriminant analysis.

As the factor indices are not normally distributed (Kolmogorov–Smirnov-Test: *p* < 0.00), non-parametric tests had to be carried out to compare the factor manifestations between the clusters. The Kruskal–Wallis *H*-Test showed that the cluster membership was significantly affected by the manifestation of factors I, II, III, V, VI, VII, and VIII (however, for factors II and III at a lower significance level only: *p* < 0.05). The test statistic for factor IV was not significant (**Table 2**), which is in line with its non-significant result in the test of equality of group means.

**Table 2:** Kruskal–Wallis *H*-Test.

Factor	*H*-Test	Degrees of freedom	Asymptotic Sig. (2-sided test)
I Achievement and Ambition	15.26	2	0.000**
II Mood Management	8.65	2	0.013
III Ability and Expertise	6.26	2	0.044
IV Technique and Information	3.23	2	0.199
V Dance	15.12	2	0.001*
VI Rhythm and Movement	37.28	2	0.000**
VII Spiritual Experiences	43.37	2	0.000**
VIII Community and Communication	15.98	2	0.000**

*p < 0.01, **p < 0.0001.

Pairwise comparisons with the Mann–Whitney *U*-Test (**Table 3**) showed significant differences for 12 of the 21 2-cluster-comparisons for all the factors that contributed significantly to the cluster membership. Factor IV does not explain differences between the clusters, and factor III was only significant at a lower level (*p* < 0.05) for the combination of clusters 1 and 3, albeit with a low effect size. Effect sizes for the significant factor influences were all above the 0.3 threshold for medium effects; factors VI and VII had effect sizes above the 0.5 threshold for large effects in two cases each. In **Figure 1**, the manifestation of the factor indices illustrates these differences. A high value indicates higher agreement with the factor.

**Table 3:** Mann–Whitney *U*-Test.

	Cluster combinations					
Factor	1–2		1–3		2–3	
	Significance level *p*	Effect size *r*	Significance level *p*	Effect Size *r*	Significance level *p*	Effect size *r*
I Achievement and Ambition	0.000**	-0.43	0.205	-0.15	0.001*	-0.37
II Mood Management	0.004*	-0.34	0.204	-0.15	0.062	-0.22
in Ability and Expertise	0.066	-0.22	0.026	-0.26	0.624	-0.06
IV Technique and Information	n. a.	n. a.	n. a.	n. a.	n. a.	n. a.
V Dance	0.707	-0.04	0.001*	-0.39	0.001*	-0.40
VI Rhythm and Movement	0.000**	-0.59	0.301	-0.12	0.000**	-0.62
VII Spiritual Experiences	0.000**	-0.63	0.000**	-0.59	0.001*	-0.39
VIII Community and Communication	0.000**	-0.43	0.000**	-0.34	0.318	-0.12

*p < 0.01, **p < 0.0001; n. a. = not applicable; *r* > 0.3, *r* > 0.5.

As the criteria for Pearson’s chi-square test were not met (more than 20% of the cells had expected counts of less than 5), a Kolmogorov–Smirnov test was carried out to examine any sociodemographic differences between the clusters. The Kolmogorov-Smirnov test did not show any significant differences regarding gender, age, main instrument, age at which the student began to play an instrument, parental musical expertise, level of studies, or career goals (*p* > 0.05). The clusters only differed significantly regarding the grouped practice hours (1–2, 2.5–4, and 4.1–7) [*H*(2) = 6.12, *p* = 0.047]. Mann–Whitney *U*-Tests were run to test which cluster combinations differed regarding the practice hours per day. Students in cluster 1 practiced significantly more hours per day than students in cluster 2 (*U* = 137, *z* = -2.45, *p* = 0.14, *r* = 0.05). However, the effect size *r* is very low and the differences therefore negligible. In the discussion we will nevertheless refer to the clusters’ sociodemographic tendencies compared across all three clusters, or within one cluster (**Table 4**).

**Table 4:** Sociodemographic tendencies of the clusters.

	Cluster 1	Cluster 2	Cluster 3
Gender	-	Male ♢	Female
Age	>24 ♢	-	≤20
Main instrument	Western instrument voice ♢	Chinese instrument	Western instrument
Age at start of practice (main instrument)	≤6 and 11+ □	≤6 and 7–10	≤6
Practice hours per day	2.6–4 h □	≤2.5 h	≤2.5 and 2.6–4 h
Parental instrumental activity (past or present)	None/one parent □	None	None
Current level of studies	Postgraduates ♢	Undergraduates	1st and 2nd year undergraduates
Career goal	Music teacher □	Music teacher/performer ♢/other ♢	Music teacher/performer ♢

*♢ = dominant value(s) of the characteristic compared across the clusters.*

*□ = dominant value(s) of a characteristic compared within one cluster and across the clusters.*

*No symbol attached = dominant value(s) of the characteristic compared within one cluster.*

## Discussion

The first part of the discussion will address the eight factors of the MUSCI-CN scales regarding their item composition and meaning. Second, the clusters will be interpreted according to their factor manifestations, along with an explanation of the importance of the factors for cluster membership. Thereafter, the results will be compared to Spychiger et al.’s (2010) work, and the relevance of the current findings will be discussed. Finally, the study’s limitations are addressed, and further research desiderata are highlighted.

### The Eight Factors of the MUSCI-CN

Factor I **Achievement and Ambition** is the strongest factor, with 11 items. It includes items of the original MUSCI from the scales *Ideal Musical Self*, *Musical Ability*, *Musical Communication*, and *Performance and Ambition*. Although it might seem that this new factor does not resemble the original scale, the items all point in the same direction. The items from the scale *Ideal Musical Self* resemble the included items from the scales *Performance and Ambition* and *Musical Ability* through the striving for high musical achievement. This focus on ambitious achievement and an ideal musical-self characterizes these items in contrast to those items of the *Musical Ability* scale which fell into the new factor III **Ability and Expertise**. Surprisingly, item 53 “I strive toward high musical achievement,” which explicitly dealt with this striving from the scale *Performance and Ambition* could not be retained. However, item 47 “I love applause,” from the scale *Musical Communication* fell into factor I which can, for example, be explained through the motivation to achieve and deserve applause. Although this factor is composed of three of the MUSCI scales, it is coherent in itself in emphasizing the importance of achievement and ambition. The significance of this factor might partly be explained by the emphasis on effort and self-enhancement in Chinese education (e.g., Huang, 2012). Indeed, discussions of talent in Chinese society do not focus on a potential (inborn) ability, but rather on the belief in an individual’s effort to achieve.

Factor II **Mood Management** reproduced the MUSCI scale *Mood Management* very well, as five of the six items where retained. Only item 26 “Music helps me to diminish anger,” was not included. This might be explained by the Chinese value of social harmony and the tendency toward emotional regulation (Bond, 1993) so that the students did not want to point to a negatively connoted emotion.

Factor III **Ability and Expertise** consists of items from the MUSCI scale *Musical Ability*. The items emphasize current ability and the individual’s existing musical (above average) expertise (this is in contrast to the items from factor I which tend to be more future- or goal-oriented). However, the factor **Ability and Expertise** of MUSCI-CN seems to be of lower importance than the MUSCI scale *Musical Ability*, which formerly was the strongest factor, encompassing 10 items. The striving for higher skills and self-enhancement seen in the most important factor—factor I—may be linked to the high value traditionally placed on Confucian ideals of self-cultivation and high achievement. The Chinese students might therefore have put more emphasis on development and effort than Spychiger’s Western samples, who might have emphasized the ability aspect.

Factor IV **Technique and Information** also mirrored the MUSCI scale, as it consisted of three of the four items from the eponymous scale (with the above-mentioned limitations regarding its importance). The items deal with an interest in the functionality of musical instruments and the production and editing of music. Only the item about music playing devices could not be retained. This is the item which is not directly related to an active production of music. It might be that the students did not relate technical music playing devices to their musical activity and therefore gave their interest in these devices a lower rating. Moreover, it is possible that the wording “musical playing devices” was not understood well or was not specific enough in its Chinese translation.

The original MUSCI scale *Movement and Dance* could on one hand be reproduced as all items could be retained in the MUSCI-CN. On the other hand, the scale split into two factors in our study, namely the factors V **Dance** and VI **Rhythm and Movement**. Dance includes all items of the MUSCI scale which directly deal with active dancing, while the **Rhythm and Movement** items point to the probability and ease with which someone moves to the rhythm of music. The MUSCI scale *Movement and Dance* could therefore not only be successfully confirmed regarding its content, but could even be refined. Whether such a distinction can be maintained in further studies in China or in a Western context has to be examined. It would, furthermore, be interesting to test this scale with participants who have a background in dance, musicals, or theater.

Factor VII **Spiritual Experiences** encompasses two of the four items of the MUSCI scale of the same name. They point to the importance of music (in the form of prayer) to get in contact with or feel the divine. The two items of the original MUSCI scale *Spiritual Experiences* which did not become part of factor VII might have attributed too much power to making music and enabling spiritual experiences or change in people. It is conceivable that the Chinese students did not attribute such high influence to their playing or singing as they see themselves as learners (cf. self-cultivation) who cannot yet claim such skills, which still reside in their teachers only. Moreover, the MUSCI scale *Spirituality* for musicians and non-musicians could not be confirmed by the principal component analysis. Despite the name Spirituality, the items in this original scale basically also dealt with spiritual experiences, however, not in connection with one’s own music making. Therefore, these broader items may have been too far away from the students’ lifeworld. The items could therefore have been problematic at a general level or because of their wording. This interesting question has to be further explored in other studies, as we could not elaborate on a possible connection of (different kinds of) spirituality and music in this investigation.

The eighth and last factor **Community and Communication** of the MUSCI-CN contains one item each from the MUSCI scales *Community* and *Musical Communication*. The two items link music-making and musical events with meeting and getting in contact with other people. The other items from the original scales were even more focused on socializing (*Community* scale) or on performing and being part of an ensemble (*Musical Communication* scale), but could not be confirmed by our sample. This community/communication aspect must be further assessed. Here we might have encountered the same problem as with factor VII **Spiritual Experiences**, which did not include items that point to a possible power or effect of the students’ own music making.

### Characterization of the Clusters^4^

Cluster 1 is characterized by a considerably high agreement with **Achievement and Ambition**. This agreement is significantly higher than in cluster 2 and similar to that of cluster 3. The same pattern of cluster differences was found for **Rhythm and Movement**. Of particular note is the very low importance of this factor for cluster 2. Persons in cluster 1 also agree significantly more with statements under **Mood Management** than those in cluster 2 but there was no significant difference found between them and cluster 3. Neither could clusters 2 and 3 be differentiated regarding **Mood Management**. **Dance** was of significantly less importance for clusters 1 and 2—which did not differ significantly from each other—than for cluster 3. **Spiritual Experiences**, however, was significantly distinct in all three clusters. It was of high importance for cluster 1, of middle importance for cluster 3, and of no importance for cluster 2. Especially striking is the most significant high value of this factor for cluster 1. **Community and Communication** is of significantly high importance for cluster 1 in contrast to clusters 2 and 3, which did not differ considerably from each other in their low agreement with this factor. **Ability and Expertise** only differentiated clusters 1 and 3, albeit with a low effect size, with cluster 1 putting more importance on this factor. These homogeneous factor values are hardly surprising, as our sample consisted of music students from one university who are more similar in their (perceived) musical ability and expertise than musicians and non-musicians of different backgrounds whose musical self-concepts were assessed in Spychiger et al.’s (2010) study. Neither did **Technique and Information** distinguish between the clusters, which is reflected in Spychiger et al.’s (2010) results as this factor had already shown some instability and further investigations might even eliminate the corresponding items.

Regarding the sociodemographic attributes, no significant differences between the clusters could be ascertained, which underlines the need for follow-up studies with larger sample sizes and participants from more diverse backgrounds. Nevertheless, some sociodemographic tendencies could be found (**Table 4**) and are depicted in the subsequent cluster characterization.

These cluster descriptions must be interpreted with the utmost caution as there is a variety of characteristics associated with the clusters which must be re-assessed in further studies. With the present data we can only suspect some connections and only speculate on reasons for the factor manifestations and sociodemographic patterns. The need for such cautious interpretation is discussed further in the subsequent sections, which address the study’s limitations.

#### Cluster 1 – “Motivated Achievers”

Persons in cluster 1 appear to have high ambitions and strive for higher musicianship and musical skills. They think they can use music to influence their mood, and that their musical ability and expertise is above average. They seem to have a certain interest in technical aspects of music making, editing and producing. While they appear not to like and rather avoid dancing, they easily move to the rhythm of music. Also, they seem to be able to use music to enable spiritual experiences and connect with people.

People in this cluster tend to be older than those in the other clusters (two third of all participants who are 24 years or older are in cluster 1) and studying a Western instrument or voice as main instrument. Most of the students in this cluster started their instrumental training before or at 6 years old. However, of those who started to play their main instrument relatively late (11+ years), the majority are in cluster 1. The late start aligns with the relatively high number of singers in this cluster as they might have started their training later than instrumentalists such as pianists or violinists. Persons in cluster 1 tend to practice between 2.6 and 4 h per day (and significantly longer than persons in cluster 2). This motivation to practice is mirrored in the high value they placed on the factor Achievement and Ambition. Furthermore, half of the individuals who already completed the Bachelor’s degree (postgraduate students) are in cluster 1. Persons in this cluster therefore seem to be not only slightly older but also more experienced than students in the other two clusters. More than half of the persons in cluster 1 had no parent and one third only one parent who plays/played an instrument. Of all the participants with one parent playing an instrument, the majority was assigned to cluster 1. Additionally, three quarters of the students in cluster 1 aspire to a teaching career, which seems curious in light of their high achievement motivation. On the other hand, it might be seen as a plausible choice if only those who think of themselves as musically competent can picture themselves as mediators of musical knowledge and skills.

In sum, a student in cluster 1 could be characterized as the achieving and abled, mood-managing, spiritual and socializing musician, who will not dance but move to the rhythm of music. Rhythm might be perceived as an important factor for understanding and feeling music, while dance might not be directly related to (classical) concert music; dancing might even be perceived as a less worthwhile activity than instrumental musical activity. In contrast, spiritual experiences are maybe interpreted as moments where music is above the musician and other things, corresponding to the attributed power of music in the social and communicative regard. This cluster therefore seems to rate the value and effects of music quite highly, which in turn could lead to high ambitions and consequently to increased achievement motivation and skill development (Wigfield and Eccles, 2000)—which ultimately leads to experience of success.

#### Cluster 2 – “Nay-Sayers”

Persons in cluster 2 seem to have comparatively low musical ambitions and less striving for achievement. They also do not (or cannot) use music to manage their mood, and their perceived ability and expertise is below average. Although factor IV Technique and Information does not significantly differentiate the clusters, cluster 2 visibly has the lowest value. Persons in this cluster also seem not to like dancing and they even more strongly oppose the statements regarding moving to rhythm. The same picture is repeated concerning spiritual experiences; cluster 2 seems to negate using music as a kind or prayer or as a mediator to feel the divine. Furthermore, persons in this cluster tend not to relate musical activity to social or communicative aspects.

Although females predominate in all clusters, cluster 2 is the one with the highest proportion of males compared to females per cluster. Compared within the cluster, a tendency to play a Chinese instrument can be noted, while the start of the instrumental training seemed to occur before the age of 11. The practice hours of persons in cluster 2 are significantly lower than those of persons in cluster 1. The vast majority in cluster 2 seems to practice no more than 2.5 h per day, and no one more than 4 h. Moreover, most of the cluster 2 students’ parents did not play an instrument; a higher share than in clusters 1 and 3. Within cluster 2, about one third of the persons are postgraduate students, which is more than within the other clusters (while *across* all clusters most of those who have completed a Bachelor’s degree/are postgraduates where assigned to cluster 1). Finally, the career goal is not as clear as in cluster 1. Although teaching was the number one career goal in all clusters, persons in cluster 2 more likely strive for a career in other areas than teaching and performing when “another career goal” is compared across the clusters. This result might be seen in light of the rather low achievement motivation and the low practice hours per day. Perhaps a low practice motivation and the disinterest in a career as musician are related, although the direction of this relation cannot be determined: does a low motivation hinder the possibility of working as musician or do the low chances of success undermine the motivation?

In sum, persons in cluster 2 can be seen as nay-sayers in the sense of a rather strong disagreement with most of the assessed aspects related to music making and musical experiences. This might be explained by their low values on the achievement and ability factors. Someone who does not perceive him- or herself as skilled and abled in a domain is more likely to lower his or her achievement expectations and consequently might deny the effects that products of this domain can have on other aspects of life, like the effect of music on spiritual or social experiences. Persons in this cluster also do not seem to dance and move to rhythm naturally. A perceived lower ability in dancing and feeling rhythms might, for example, be part of a lower ability and achievement motivation in music more generally.

#### Cluster 3 – “Young Dreamers”

For persons in cluster 3 ambitions and achievement appear to be important and they use music for mood management. They perceive their ability and expertise as well as their interest in technical aspects of music making as average (compared to all clusters). Significantly important for people in this cluster is the rhythmic (or perhaps even kinaesthetic) aspect of music as they like to dance and especially value movement (and easily can move) to rhythm. They do not seem to relate music to spiritual experiences (although they do not oppose it as strongly as cluster 2), and they also do not seem to put much emphasis on communicative and social aspects of music.

Persons in cluster 3 tend to be younger than 20 years old, with none being older than 24. A comparison of the distribution of males across the clusters shows males to be rather underrepresented in this cluster. The majority of persons in this cluster play a Western instrument and these students tend to have had an early beginning to their instrumental training: half of the persons in this cluster started to play their main instrument at 6 years or younger, and a third between 7 and 10 years. In contrast to persons in cluster 1 who tended to practice 2.6–4 h per day and in cluster 2 who had a tendency to practice 2.5 h per day or less, persons in cluster 3 practice for 1–4 h per day. The picture regarding parents playing an instrument resembles that of cluster 1: almost two thirds of the students in cluster 3 had no parents playing an instrument. Furthermore, they tended to be Bachelor’s students in the first 2 years of their studies. More than two thirds of the persons assigned to cluster 3 are aiming for a teaching career, but almost one third aim for a career as a performer. Of all participants aiming for a performing career more than half have been assigned to cluster 3. Other career options apart from teaching and performing do not seem to be of importance in this cluster.

In sum, cluster 3 might be characterized as dancers and rhythmists with average achievement motivation and perceived musical skills. As their link between spiritual experiences and music does not seem to be very strong and they do not appear to emphasize the importance of socializing and communicating through music, it might be assumed that they do not attribute as much influence or power to music as persons in cluster 1, whose perceived ability and expertise also seem to be higher. Yet, the wish for a career as a performer and the perceived average musical abilities would appear to contradict each other. Together with the younger age of this cluster, it might be assumed that older students (of other clusters and especially of cluster 1) perhaps assess the likeliness of a performing career more realistically and opt for other career paths, whereas this insight has yet to be developed by students in cluster 3.

### MUSCI and MUSCI-CN in Comparison

A comparison of the self-concept clusters to the clusters measured by Spychiger et al. (2010) is difficult, as Spychiger also included non-musicians in the sample and had a broader data foundation, which resulted in the following six musical self-concept types: ideal-oriented music expert, mood music user, professional musician, couch potato, party lover, secluded music individualist (translation by the authors). Cluster 2 can be compared to the “couch potatoes,” since they evaluated their abilities as below average, they opposed to dancing and moving to musical rhythms, and they did not use music for mood regulation. The “couch potatoes” tended to be male, which is mirrored in a slight tendency in cluster 2 as well. In Spychiger’s study these participants did not make or study music, but identified as music listeners. Clusters 1 and 3, however, do not show notable similarities with Spychiger’s results across several factors—which of course are not exactly the same for both samples.

These divergent findings do not necessarily indicate the invalidity of Spychiger’s findings or the results of our explorative study. It was to be expected that the results would differ if the MUSCI was applied in a translated version in another cultural setting and with music students instead of a more diverse sample. It would have been too big a step to go from the application of the MUSCI in a German setting to a Chinese setting without a certain restriction of the sample; this restriction allowed for better control of the variables especially regarding sociodemographic features. However, it probably also caused the similarity of the clusters in the area of sociodemographic characteristics. Consequently, the inclusion of a second, independent sample to test the resulting model would have been desirable and must be aimed for in further studies.

The limited number of items of the MUSCI-CN factors VI Rhythm and Movement, VII Spiritual Experiences, and VIII Community and Communication also seems problematic, as more than two items tend to better represent a construct (Eisinga et al., 2012). Further applications of these factors should assess the questionnaire quality with additional items as Spychiger et al. (2010) also suggested regarding the MUSCI scales Adaptive Musical Self, Community, and Spirituality.

As the principal component analysis was carried out with all items in the present study, the focus on musicians and musicians-to-be led to an adaptation of the MUSCI scales so that three of the eight MUSCI-CN factors appear at first only suitable for musicians (bearing in mind that only six of those eight factors were significantly differentiated between the clusters):

– The factor Achievement and Ambition would be suitable for non-musicians without item 44 “I am capable of achieving the musical goals that I have set.” Cronbach’s Alpha would remain high with a value of 0.913 instead of 0.915.
– The factor Spiritual Experiences of the MUSCI-CN, however, would not be applicable to non-musicians. As stated earlier, and although the factor significantly differentiated the clusters, it is problematic regarding its content. Spirituality is very differently perceived in a Chinese compared to a Western setting and the understanding of this factor and the comparability with a Western sample has to be assessed. Qualitative interviews and focus group discussions could be helpful in clarifying how the Chinese music students link music to spirituality in general, and how spirituality and spiritual experiences are understood in the context of the musical self-concept assessment in particular.
– The factor Community and Communication can also not be presented to non-musicians in its current form, since item 43 “I play music in order to communicate with other people” can only be answered by persons who make music. As stated above, this factor faced similar problems as the spirituality factor due to the different perception and attributed importance of social and communicative aspects of music in an Asian or a Western (or collectivist vs. individualistic) society. Here, the inclusion of a qualitative approach and a methodological triangulation could also prove useful and must be considered in additional studies.

A further step would be an application of the MUSCI with a Chinese sample of non-musicians. This could shed light on the factor structure for non-musicians and improve the musical self-concept scales. Such a further cross-cultural application promises an advancement of the concept, but bears challenges, as our study shows. In the concluding section we discuss some methodological difficulties which complicate the interpretation and restrict the significance of our results.

### Methods Discussion, Limitations, and Implications

Self-concept research in a culturally specific environment encounters several stumbling blocks as, for example, Yeung (2005) pointed out. First of all, it is vital to choose the right measurement according to the hierarchy level of the self-concept in question, and there is a need for research on the assessment of the hierarchically lower subdomains or dimensions of a self-concept. As the goal of our study was to assess the general musical self-concept, the use of the MUSCI can be seen as appropriate as it does not focus on one specific component (e.g., abilities) but rather brings together several aspects of the musical self-concept.

Perhaps even more important in attempting to use the MUSCI in a Chinese setting is the appropriateness of the measuring tool for a specific sample. This concerns our questionnaire and its wording in Chinese. The translations and adaptations were conducted by a team of Chinese and Western researchers and were continually compared to the original German and English phrasing. However, as cultural concepts are embedded in languages and specific meanings cannot always be translated into another language straightforwardly (Filep, 2009), it can be assumed that some notions, phrases, and words have differing meanings for Chinese and Western students. In this regard, the reference-group-effect must also be considered. Because the reference group people compare themselves with is not the same in different cultures, cross-cultural comparisons can yield misleading results. People interpret statements (or items) based on their cultural background and the meaning of phrases or concepts in their culture. These are not necessarily—perhaps even only rarely—the same (Heine et al., 2002). In combination with the translation biases, this effect can compromise research on the musical self-concept in Asian countries when using scales that were developed and tested in the West. Thus further studies in China must be conducted in order to reassess the MUSCI-CN as well as the factors and items, in order to improve the item quality. A discussion of the questionnaire and its items in one-on-one-interviews or focus groups could be a useful approach for obtaining a Chinese wording and a comprehension of the wording which is based on an even larger common understanding. In this context, follow-up validation studies—possibly with a triangulation of qualitative and quantitative methods—might help overcome limitations of the study such as the provisional status of the current language version, which consequently limit the validity of the results and conclusions.

Another possible influence on the results is social desirability. Chinese culture values keeping face and respecting superiors such as teachers (e.g., Jin and Cortazzi, 1998). This might, for example, have led to the exclusion of items that imparted a certain power to the students’ own musical activity. The students’ modesty with respect to their self-perception as learners might have influenced their item ratings. Conversely, the factor Achievement and Ambition, with items dealing with the adaptation of the self or the musical skills, is now the strongest factor on the MUSCI-CN. This finding is in line with the social desirability and importance of self-cultivation in Confucian heritage cultures (Wang, 2013). However, the relationship between cultural backgrounds and social desirability tendencies must be examined more closely as, to date, there has been no study of this topic with regard to musical self-concept research (with regard to physical disabilities see Tam, 1995).

## Conclusion

Collectivist traditions and Confucianism cannot be seen as sole and indisputable influences on Chinese learners (Wang, 2013) and their musical self-concept. Changes are occurring in China, and Western values are gaining in importance, especially for younger generations (e.g., Zhang and Shavitt, 2003). For example, there are indicators that learning style, motivation, or student-teacher relationships are, to a certain extent, similar between Western and Chinese (music) students (Littlewood, 2000; Brand, 2001). There are, nevertheless, differences between the musical self-concepts in the different environments, as the present study indicates.

Challenges of interpretation point to the necessity of referencing overall cultural values when dealing with cross-cultural studies and using analytical dichotomies like “China” and the “West.” But they also point to opportunities offered by the cross-cultural application of a concept that can shed light on similarities or differences of cultural values. The MUSCI represents a tool for examining these assumed values, as they are mirrored in an individual’s self-concept. Therefore, as the present data can only provide preliminary conclusions, further validation studies of the MUSCI and MUSCI-CN, including a comparative perspective and application in varying cultural settings, remain topics for future research.

## Author Contributions

SP and M-AC were responsible for the conception and design of the work and data acquisition. SP analysed and interpreted the data, drafted and revised the paper, gave final approval of the version to be published and agrees to be accountable for all aspects of the work. M-AC revised the paper critically for important intellectual content, gave final approval of the version to be published and agrees to be accountable for all aspects of the work.

## Conflict of Interest Statement

The authors declare that the research was conducted in the absence of any commercial or financial relationships that could be construed as a potential conflict of interest.

## Footnotes

1 Regarding cultural influences on talent, see for example Hernández de Hahn (2002) or Ambrose (2009).

2 Hargreaves et al. (2002) presented a differentiation of musical self-perceptions or identities, namely *identities in music* (IIM) and *music in identities* (MII). The IIM relates to how we see ourselves on a general level with regard to music, for example in the culturally influenced roles as performer or listener, while MII points to the role of music as one facet of our self.

3 For (Western) research dealing with the self-concept and/or the (possible) change of self-concept and self-image from music students and/or performers to music teachers, see for example Hargreaves and Marshall (2003), Hargreaves et al. (2007), Pellegrino (2009), Ballantyne et al. (2012), and Freer and Bennett (2012). The latter particularly point to the relation of the teachers’ and pupils’ musical identity and the similar context in which they emerge. Regarding the socialization of music teachers, see for example Woodford (2002) or Isbell (2008, 2014). Niessen (2007) addressed the pedagogical, teaching-related self-concept in relation to an ability-focused musical self-concept of music teachers. A review of research on teachers’ professional identity was published by Beijaard et al. (2004). From a Chinese perspective, a change in music teachers’ development from getting familiar with to professionalizing teaching was for example explored by Chen (2011). However, further research will be needed to closer examine the link between teachers’ and pupils’ musical self-concepts; a question which cannot be answered by our survey.

4 Cf. **Table 3** for the statistics.

## References

[B1] Ambrose D. (2009). “Large-scale socioeconomic, political, and cultural influences on giftedness and talent,” in *International Handbook on Giftedness*, ed. Shavinina L. V. (Amsterdam: Springer), 885–903.

[B2] Ballantyne J. Kerchner J. L. Aróstegui J. L. (2012). Developing music teacher identities: an international multi-site study. *Int. J. Music Educ.* 30 211–226. 10.1177/0255761411433720

[B3] Beijaard D. Meijer P. C. Verloop N. (2004). Reconsidering research on teachers’ professional identity. *Teach. Teacher Educ.* 20 107–128. 10.1016/j.tate.2003.07.001

[B4] Bond M. H. (1993). Emotions and their expression in Chinese culture. *J. Nonverb. Behav.* 17 245–262. 10.1007/BF00987240

[B5] Brand M. (2001). Chinese and american music majors: cross-cultural comparisons in motivation and strategies for learning and studying. *Psychol. Music* 29 170–178. 10.1177/0305735601292006

[B6] Brand M. (2004). Collectivistic versus individualistic cultures: a comparison of American, Australian and Chinese music education students’ self-esteem. *Music Educ. Res.* 6 57–66. 10.1080/1461380032000182830

[B7] Buchborn T. Painsi M. (2010). The interdependency between musical behavior and musical self-concept. *Paper Presented at the Annual Meeting of the ISME World Conference and Commission Seminars, China Conservatory of Music (CC) and Chinese National Convention Centre (CNCC)*, Beijing.

[B8] Chen P. (2011). *The Path for Music Teachers’ Professional Development*. *Educ. Music* 11 7–8.

[B9] Chen X. (2000). “Growing up in a collectivist culture: Socialization and socioemotional development in Chinese children,” in *International Perspectives on Human Development*, eds Comunian A. L. Gielen U. P. (Lengerich: Pabst Science Publishers), 331–353.

[B10] Eisinga R. te Grotenhuis M. Pelzer B. (2012). The reliability of a two-item scale: pearson, cronbach, or spearman-brown? *Int. J. Public Health* 58 637–642. 10.1007/s00038-012-0416-3 23089674

[B11] Filep B. (2009). Interview and translation strategies: coping with multilingual settings and data. *Soc. Geogr.* 4 59–70. 10.5194/sg-4-59-2009

[B12] Freer P. K. Bennett D. (2012). Developing musical and educational identities in university music students. *Music Educ. Res.* 14 265–284. 10.1080/14613808.2012.712507

[B13] Garbati J. F. Rothschild N. (2016). Lasting impact of study abroad experiences: a collaborative autoethnography. *Forum Qual. Soc. Res.* 17.

[B14] Hallam S. (2011). “Culture, musicality, and musical expertise,” in *A Cultural Psychology of Music Education*, ed. Barrett M. S. (New York: Oxford University Press).

[B15] Hargreaves D. J. Marshall N. A. (2003). Developing identities in music education. *Music Educ. Res.* 5 263–273. 10.1080/1461380032000126355

[B16] Hargreaves D. J. Marshall N. A. North A. C. (2003). Music education in the twenty-first century: a psychological perspective. *Br. J. Music Educ.* 20 147–163. 10.1017/S0265051703005357

[B17] Hargreaves D. J. Miell D. MacDonald R. A. R. (2002). “What are musical identities, and why are they important,” in *Musical Identities*, eds MacDonald R. A. R. Hargreaves D. J. Miell D. (Oxford: Oxford University Press), 1–20.

[B18] Hargreaves D. J. Purves R. M. Welch G. F. Marshall N. A. (2007). Developing identities and attitudes in musicians and classroom music teachers. *Br. J. Educ. Psychol.* 77 665–682. 10.1348/000709906X154676 17908381

[B19] Heine S. J. (2001). Self as cultural product: an examination of east asian and North American selves. *J. Pers.* 69 881–905. 10.1111/1467-6494.696168 11767822

[B20] Heine S. J. Lehman D. R. Peng K. Greenholtz J. (2002). What’s wrong with cross-cultural comparisons of subjective likert scales? The reference-group effect. *J. Personal. Soc. Psychol.* 82 903–918. 10.1037/0022-3514.82.6.903 12051579

[B21] Hernández de Hahn J. (2002). “Cross-cultural studies in gifted education,” in *International Handbook of Research and Development of Giftedness and Talent*, 2nd Edn, eds Heller K. A. Mönks F. J. Passow A. H. (Oxford: Elsevier), 549–561.

[B22] Ho W. Law W. (2004). Values, music and education in China. *Music Educ. Res.* 6 149–167. 10.1080/1461380042000222564

[B23] Huang H. (2012). Why Chinese people play Western classical music: transcultural roots of music philosophy. *Int. J. Music Educ.* 30 161–176. 10.1177/0255761411420955

[B24] Isbell D. S. (2008). Musicians and teachers. the socialization and occupational identity of preservice music teachers. *J. Res. Music Educ.* 56 162–178. 10.1177/0022429408322853

[B25] Isbell D. S. (2014). The socialization of music teachers. a review of the literature. *Appl. Res. Music Educ.* 34 5–12. 10.1177/8755123314547912

[B26] Jin L. Cortazzi M. (1998). Dimensions of dialogue: large classes in China. *Int. J. Educ. Res.* 29 739–761. 10.1016/S0883-0355(98)00061-5

[B27] Kline R. B. (1998). *Principles and Practice of Structural Equation Modeling.* New York, NY: Guilford Press.

[B28] Li J. (2005). Mind or virtue. western and chinese beliefs about learning. *Curr. Dir. Psychol. Sci.* 14 190–194. 10.1111/j.0963-7214.2005.00362.x

[B29] Littlewood W. (2000). Do Asian students really want to listen and obey? *ELT J.* 54 31–36. 10.1093/elt/54.1.31

[B30] Marsh H. W. (1993). “Academic self-concept: theory, measurement, and research,” in *Psychological Perspectives on the Self, The Self in Social Perspective* Vol. 4 ed. Suls J. (Hillsdale, NJ: Erlbaum), 59–98.

[B31] Marsh H. W. Hau K.-T. (2004). Explaining paradoxical relations between academic self-concepts and achievements: cross-cultural generalizability of the internal/external frame of reference predictions across 26 countries. *J. Educ. Psychol.* 96 56–67. 10.1037/0022-0663.96.1.56

[B32] Marsh H. W. Martin A. J. (2011). Academic self-concept and academic achievement: relations and causal ordering. *Br. J. Educ. Psychol.* 81 59–77. 10.1348/000709910X503501 21391964

[B33] Marsh H. W. Shavelson R. (1985). Self-concept: its multifaceted, hierarchical structure. *Educ. Psychol.* 20 107–123. 10.1207/s15326985ep2003_1

[B34] McInerney D. M. Cheng R. W. Mok M. M. C. Lam A. K. H. (2012). Academic self-concept and learning strategies direction of effect on student academic achievement. *J. Adv. Acad.* 23 249–269. 10.1177/1932202X12451020

[B35] Morin A. J. S. Scalas L. F. Vispoel W. Marsh H. W. Wen Z. (2015). The music self-perception inventory: development of a short form. *Psychol. Music* 1–20. 10.1177/0305735615592690

[B36] Niessen A. (2007). Musikalische und pädagogische selbstkonzepte von musiklehrerinnen und musiklehrern. *Diskussion Musikpädagogik* 33 30–40.

[B37] Oyserman D. Elmore K. Smith G. (2011). “Self, self-concept, and identity,” in *Handbook of Self and Identity*, 2nd Edn, eds Leary M. R. Tangney J. P. (New York, NY: Guilford Press), 69–104.

[B38] Pellegrino K. (2009). Connections between performer and teacher identities in music teachers: setting an agenda for research. *J. Music Teacher Educ.* 19 39–55. 10.1177/1057083709343908

[B39] Petersen S. Camp M.-A. (2015). “The musical self-concept of Chinese music students,” in *Proceedings of the Ninth Triennial Conference of the European Society for the Cognitive Sciences of Music (ESCOM)*, Manchester, 654–655.

[B40] Phillipson S. N. (2013). “Confucianism, learning self-concept and the development of exceptionality,” in *Exceptionality in East Asia: Explorations in the Actiotope Model of Giftedness*, eds Phillipson S. N. Stoeger H. Ziegler A. (New York, NY: Routledge), 40–64.

[B41] Ruismäki H. Tereska T. (2006). Early childhood musical experiences: contributing to pre-service elementary teachers’ self-concept in music and success in music education (during student age). *Euro. Early Childhood Educ. Res. J.* 14 113–130. 10.1080/13502930685209841

[B42] Sanders P. D. Browne L. A. (1998). Music self-concept of non-music majors. *Contrib. Music Educ.* 25 74–86.

[B43] Shavelson R. J. Hubner J. J. Stanton G. C. (1976). Self-concept: validation of construct interpretations. *Rev. Educ. Res.* 46 407–441. 10.3102/00346543046003407

[B44] Spychiger M. (2012). Das musikalische Selbstkonzept. *Schwerpunktthema Wissenschaft - Kunst - Forschung* 12 45–49.

[B45] Spychiger M. (in press). “Musical identity and musical self-concept,” in *The Oxford Handbook on Musical Identity*, eds Hargreaves D. J. MacDonald R. Miell D. (Oxford: Oxford University Press).

[B46] Spychiger M. Gruber L. Olbertz F. (2009). “Musical self-concept presentation of a multi-dimensional model and its empirical analyses,” in *Proceedings of the 7th Triennial Conference of European Society for the Cognitive Sciences of Music (ESCOM)*, eds Louhivuori J. Eerola T. Saarikallio T. Eerola P.-S. (Jyväskylä: European Society for the Cognitive Sciences of Music), 503–506.

[B47] Spychiger M. Olbertz F. Gruber L. (2010). *Das musikalische Selbstkonzept. Konzeption des Konstrukts als mehrdimensionale Domäne und Entwicklung Eines Messverfahrens.* Final Scientific Report to the Swiss National Science Foundation (Beitrag Nr. 100013–116208).

[B48] Tam S.-F. (1995). Relationship of self-concept and social desirability tendency of hong kong chinese adults with physical disabilities. *Perceptual Motor Skills* 81 731–738. 10.2466/pms.1995.81.3.731 8668428

[B49] Tweed R. G. Lehman D. R. (2002). Learning considered within a cultural context: confucian and Socratic approaches. *Am. Psychol.* 57 89–99. 10.1037/0003-066X.57.2.89 11899565

[B50] Vispoel W. P. (1996). The development and validation of the arts self-perception inventory for adults. *Educ. Psychol. Measure.* 56 719–735. 10.1177/0013164496056004013

[B51] Wang J. (2013). Confucian heritage cultural background (CHCB) as a descriptor for chinese learners: the legitimacy. *Asian Soc. Sci.* 9 105–113. 10.5539/ass.v9n10p105

[B52] Wang Q. (2006). Relations of maternal style and child self-concept to autobiographical memories in Chinese, Chinese immigrant, and european american 3-year-olds. *Child Dev.* 77 1794–1809. 10.1111/j.1467-8624.2006.00974.x 17107461

[B53] Weiber R. Mühlhaus D. (2010). *Strukturgleichungsmodellierung: Eine Anwendungsorientierte Einführung in die Kausalanalyse mit Hilfe Von AMOS, SmartPLS und SPSS.* Berlin: Springer.

[B54] Wigfield A. Eccles J. S. (2000). Expectancy–value theory of achievement motivation. *Contemp. Educ. Psychol.* 25 68–81. 10.1006/ceps.1999.1015 10620382

[B55] Woodford P. (2002). “The social construction of music teacher identity in undergraduate music education majors,” in *The New Handbook of Research on Music Teaching and Learning: A Project of the Music Educators National Conference*, eds Colwell R. Richardson C. (Oxford: Oxford University Press), 675–694.

[B56] Yeung S. S. (2005). “Reconsidering the measurement of student self-concept. use and misuse in a chinese context,” in *New Frontiers for Self Research* Vol. 2 eds Marsh H. W. Craven R. McInerney D. M. (Charlotte, NC: IAP), 233–256.

[B57] Zhang J. Shavitt S. (2003). Cultural values in advertisements to the Chinese X-Generation–promoting modernity and individualism. *J. Advert.* 32 23–33. 10.1080/00913367.2003.10639047

