# Supplementary material for: The Musical Self-Concept of Chinese Music Students
Source: Front Psychol. 2016 May 27;7:776. doi: 10.3389/fpsyg.2016.00776 (PMC4882319; doi:10.3389/fpsyg.2016.00776)
Supplement: Supplementary file 1 [file Data_Sheet_1.PDF]

Supplementary Material

The Musical Self-Concept of Chinese Music Students

Suse Petersen[1]*, Marc-Antoine Camp[1]

[1]School of Music, Lucerne University of Applied Sciences and Arts, Lucerne, Switzerland

*** Correspondence:** Suse Petersen, School of Music, Lucerne University of Applied Sciences and Arts, Zentralstrasse 18, 6003 Lucerne, Switzerland.

suse.petersen@hslu.ch

1 Items of the Musical Self-Concept Inquiry (Spychiger et al., 2010).

1. Music relieves me from daily routine.
2. I am challenged to make the most of my musical ability.
3. The convivial facet in music means a lot to me.
4. For me, music is a specific source of mood management.
5. I can sing well.
6. My physical reaction to music is different from what it was formerly.
7. It occurs that music listening is a spiritual experience to me.
8. My musical ability is above average.
9. I would like to have higher musicianship.
10. I respond to the rhythm of music with movement.
11. I am interested in how musical instruments function.
12. I have the ability to teach other people about music.
13. I passionately love to dance.
14. With regards to spirituality in music, my current susceptibility is different from earlier in my life.
15. I easily hear harmonics and can sound out voices.
16. The technical options to produce music are fascinating to me.
17. Today my emotional perception of music is different from earlier in my life.
18. I would like to have a greater understanding of music.
19. I pay a lot of attention to the technical quality of my music reproduction devices.
20. Dancing satisfies my need for physical movement.
21. With music I can forget my sorrows.
22. I have no musical talent.
23. By the means of music I can get contact with God.
24. I easily socialize by the means of music.
25. Learning to play an instrument is too laborious to me.
26. Music helps me to diminish anger.
27. Spirituality in music means more to me than to other people.
28. I regret that I am not more musically creative.

29. I am concerned with the question of how music is produced.
30. I can relax with music.
31. I would like to know more about the characteristics of the different musical styles.
32. I feel that I could have become a great musician.
33. I avoid dancing since I don't dance well.
34. Music helps me to cope with stress.
35. I am an expert as regards to certain musical styles.
36. I go to music happenings in order to meet people.
37. My intellectual understanding of music has changed during the years.
38. To me, music is an expression of community more than it is to others.
39. I easily move to the rhythm of music.
40. I am not the type for spiritual experiences with music.
41. That I am musically competent means a lot to me.
42. I would like to have more knowledge of the technical features and options in music.

For Musicians

43. I play music in order to communicate with other people.
44. I am capable of achieving the musical goals that I have set.
45. I easily become part of a musical ensemble.
46. I am musically ambitious.
47. I love the applause.
48. Musical activity can alter my mood.
49. I take advantage of any opportunity in order to advance my musical ability.
50. When making music I have to be able to forget time and place.
51. For me, making music is a special kind of prayer.
52. I strive toward high musical achievement.
53. I sense that the music I perform connects people.
54. I usually play music for my self-satisfaction.

Supplementary Table 1. Item assignment of the MUSCI-CN and original scale of the items.

Factor	No.	Item	Original scale of the item in the MUSCI
I Achievement and Ambition	2	It appeals to me to make the most of my musical ability.	Musical ability
	22	I have no musical talent.	
	32	I feel that I could have become a great musician.	
	9	I would like to have higher musicianship.	Ideal musical self
	28	I regret that I am not more musically creative.	
	42	I would like to have more knowledge of the technical features and options in music.	
	44	I am capable of achieving the musical goals that I have set.	Performance and ambition
	46	I am musically ambitious.	
	49	I take advantage of any opportunity to advance my musical ability.	
	58	I am proud of my musical skills.	
	47	I love applause.	Musical communication
II Mood Management	1	Music can carry me away from everyday life.	Mood management
	4	I can purposefully influence my mood through music.	
	21	With music I can forget my sorrows.	
	30	I can relax with music.	
	34	Music helps me to cope with stress.	
III Ability and Expertise	8	My musical ability is above average.	Musical ability
	12	I have the ability to teach other people about music.	
	15	I easily hear harmonies and pick out voices.	
	35	I am an expert as regards to certain musical styles.	
VI Technique and Information	11	I am interested in how musical instruments function.	Technique and information
	16	The technical options to edit music are fascinating to me.	
	29	I am concerned with the question of how music is produced.	
V Dance	13	I passionately love to dance.	Movement and Dance
	20	Dancing satisfies my need for physical movement.	
	33	I avoid dancing since I don't dance well.	
VI Rhythm and Movement	10	When I hear music, I start to move my body to the rhythm.	Movement and Dance
	39	I easily move to the rhythm of music.	
VII Spiritual Experiences	51	For me, making music is a special kind of prayer.	Spiritual Experiences
	57	I make music in order to feel the divine.	
VIII Community and Communication	36	I go to musical events in order to meet people.	Community
	43	I play music in order to communicate with other people.	Musical communication
